# Supplementary material for: Machine Learning Inference of Gene Regulatory Networks in Developing Mimulus Seeds
Source: Plants (Basel). 2024 Nov 23;13(23):3297. doi: 10.3390/plants13233297 (PMC11644258; doi:10.3390/plants13233297)
Supplement: Supplementary file 1 [file plants-13-03297-s001.zip › Supplemental_figures.pdf]

Supplementary Figures

Genes with Highest Network Motif Scores in GRN1<br>Endosperm enriched set filtered using Modified Shannon Entropy, using RTP-STAR for inferrence.				
NMS	*M. guttatus* TOLv5.0	Total Regulations	*A. thaliana* homolog	Araport11 Description
42	MgTOL.J0773	27		
40	MgTOL.F0855	20		
33	MgTOL.I0674	21		
32	MgTOL.I0231	21	AT1G74680	Exostosin family protein
32	MgTOL.L0525	18	AT3G05270	Plant protein of unknown function (DUF869)
31	MgTOL.N1571	17	AT1G66960	Terpenoid cyclases family protein
30	MgTOL.D2250	17		
30	MgTOL.D0718	16		
21	MgTOL.F0867	14		
19	MgTOL.K0640	18		
19	MgTOL.C0807	14	AT1G20160	Subtilisin-like serine endopeptidase family protein
19	MgTOL.M1044	13		
19	MgTOL.D0312	11		
17	MgTOL.L1626	13		
15	MgTOL.N0174	16		

Figure S1 Breakdown of the most connected genes in the inferred gene regulatory network GRN1, which was inferred using RTP-STAR and the endosperm enriched, MSE filtered gene set. NMS depicts the network motif score or the number of motifs that the corresponding gene occurred in.

Genes with Highest Network Motif Scores in GRN2<br>Endosperm enriched set filtered using Modified Shannon Entropy, using KBoost for inferrence.				
NMS	*M. guttatus* TOLv5.0	Total Regulations	*A. thaliana* homolog	Araport11 Description
10	MgTOL.C0807	16	AT1G20160	Subtilisin-like serine endopeptidase family protein
8	MgTOL.J1322	10		
8	MgTOL.E0972	10		
8	MgTOL.H0098	7	AT4G25930	Protein of unknown function (DUF295)
6	MgTOL.F0292	15	AT4G25750	ABC-2 type transporter family protein
6	MgTOL.N3332	12	AT1G44350	IAA-leucine resistant (ILR)-like gene 6
6	MgTOL.I0600	12	AT5G50600	hydroxysteroid dehydrogenase 1
6	MgTOL.H0713	12		
6	MgTOL.N2817	10	AT5G25420	Xanthine/uracil/vitamin C permease
6	MgTOL.L1446	10	AT3G09530	exocyst subunit exo70 family protein H3
6	MgTOL.B1676	8		
6	MgTOL.J1894	7	AT5G06760	Late Embryogenesis Abundant 4-5
4	MgTOL.O1184	11	AT3G47380	Plant invertase/pectin methylesterase inhibitor superfamily protein
4	MgTOL.N1571	11	AT1G66960	Terpenoid cyclases family protein
4	MgTOL.H0794	10		

Figure S2 Breakdown of the most connected genes in the inferred gene regulatory network GRN2, which was inferred using KBoost and the endosperm enriched, MSE filtered gene set. NMS depicts the network motif score or the number of motifs that the corresponding gene occurred in.

Genes with Highest Network Motif Scores in GRN3<br>Expressed gene set filtered using Modified Shannon Entropy, using RTP-STAR for inferrence.				
NMS	*M. guttatus* TOLv5.0	Total Regulations	*A. thaliana* homolog	Araport11 Description
548	MgTOL.E1405	53	AT5G11540	D-arabinono-1,4-lactone oxidase family protein
498	MgTOL.H0995	47		
482	MgTOL.J1733	48		
423	MgTOL.H1205	53	AT2G32280	Protein of unknown function (DUF1218)
421	MgTOL.I0701	43	AT1G09360	Plant invertase/pectin methylesterase inhibitor superfamily protein
397	MgTOL.L1160	50		
396	MgTOL.A0833	40		
389	MgTOL.N0174	43		
388	MgTOL.J1275	45		
343	MgTOL.D1471	42	AT1G74110	cytochrome P450, family 78, subfamily A, polypeptide 10

Figure S3 Breakdown of the most connected genes in the inferred gene regulatory network GRN3, which was inferred using RTP-STAR and the MSE filtered gene set. NMS depicts the network motif score or the number of motifs that the corresponding gene occurred in.

Genes with Highest Network Motif Scores in GRN4<br>Expressed gene set filtered using Modified Shannon Entropy, using KBoost for inferrence.				
NMS	*M. guttatus* TOLv5.0	Total Regulations	*A. thaliana* homolog	Araport11 Description
7	MgTOL.N3074	17		
5	MgTOL.N2942	20	AT1G34245	Putative membrane lipoprotein
4	MgTOL.F1017	23	AT1G26797	Plant self-incompatibility protein S1 family
4	MgTOL.F0292	18	AT4G25750	ABC-2 type transporter family protein
4	MgTOL.D1747	16		
4	MgTOL.M0621	15		
4	MgTOL.O1184	12	AT3G47380	Plant invertase/pectin methylesterase inhibitor superfamily protein
4	MgTOL.H0979	12		
3	MgTOL.G0155	15	AT1G56210	Heavy metal transport/detoxification superfamily protein
3	MgTOL.E0094	15		
3	MgTOL.J1792	14		
3	MgTOL.H2416	14	AT4G29035	Plant self-incompatibility protein S1 family
3	MgTOL.F0405	14		
3	MgTOL.M0620	13		
3	MgTOL.E0175	13	AT1G10640	Pectin lyase-like superfamily protein

Figure S4 Breakdown of the most connected genes in the inferred gene regulatory network GRN4, which was inferred using KBoost and the MSE filtered gene set. NMS depicts the network motif score or the number of motifs that the corresponding gene occurred in.

Genes with Highest Network Motif Scores in GRN5<br>Annotated transcription factor network inferred using RTP-STAR.				
NMS	*M. guttatus* TOLv5.0	Total Regulations	*A. thaliana* homolog	Araport11 Description
365	MgTOL.K0844	65	AT5G16600	myb domain protein 43
365	MgTOL.C0700	57		
340	MgTOL.M1104	56	AT4G18570	Tetratricopeptide repeat (TPR)-like superfamily protein
340	MgTOL.B0360	54	AT4G34050	S-adenosyl-L-methionine-dependent methyltransferases superfamily protein
337	MgTOL.J0021	51		
305	MgTOL.B1535	51		
277	MgTOL.D0469	49	AT5G44030	cellulose synthase A4
268	MgTOL.E0683	51	AT3G61910	NAC domain protein 66
264	MgTOL.N1181	42	AT4G34050	S-adenosyl-L-methionine-dependent methyltransferases superfamily protein
260	MgTOL.M1070	48	AT4G18780	cellulose synthase family protein
233	MgTOL.D1830	31		
228	MgTOL.D2250	42		
223	MgTOL.E0960	35	AT1G24735	S-adenosyl-L-methionine-dependent methyltransferases superfamily protein
222	MgTOL.H0567	37	AT2G23760	BEL1-like homeodomain 4
209	MgTOL.M1149	44	AT4G28500	NAC domain containing protein 73

Figure S5 Breakdown of the most connected genes in the inferred gene regulatory network GRN5, which was inferred using RTP-STAR and the annotated TF gene set. NMS depicts the network motif score or the number of motifs that the corresponding gene occurred in.

Genes with Highest Network Motif Scores in GRN6<br>Annotated transcription factor network inferred using KBoost.				
NMS	*M. guttatus* TOLv5.0	Total Regulations	*A. thaliana* homolog	Araport11 Description
4	MgTOL.H1094	20		
3	MgTOL.C0462	16	AT3G61040	cytochrome P450, family 76, subfamily C, polypeptide 7
3	MgTOL.I1131	14	AT1G54180	BREVIS RADIX-like 3
3	MgTOL.K0341	13	AT1G80710	DROUGHT SENSITIVE 1
3	MgTOL.C1187	13	AT1G35660	(1 of 1) PTHR15000:SF1 - ERYTHROID DIFFERENTIATION-RELATED FACTOR 1
3	MgTOL.M0897	9		
3	MgTOL.E0261	9		
2	MgTOL.I0668	17		
2	MgTOL.B0358	17	AT1G49770	basic helix-loop-helix (bHLH) DNA-binding superfamily protein
2	MgTOL.D1976	16	AT1G07370	proliferating cellular nuclear antigen 1
2	MgTOL.N0954	15		
2	MgTOL.F0366	15	AT5G23750	Remorin family protein
2	MgTOL.N0529	14	AT1G55500	evolutionarily conserved C-terminal region 4
2	MgTOL.D1069	14		
2	MgTOL.K0797	13	AT4G37180	Homeodomain-like superfamily protein

Figure S6 Breakdown of the most connected genes in the inferred gene regulatory network GRN6, which was inferred using KBoost and the annotated TF gene set. NMS depicts the network motif score or the number of motifs that the corresponding gene occurred in.
